# Long-Term Creep and Shrinkage Behavior of Concrete-Filled Steel Tube

**DOI:** 10.3390/ma14020295

**Published:** 2021-01-08

**Authors:** Doan-Binh Nguyen, Wei-Sheng Lin, Wen-Cheng Liao

**Affiliations:** Department of Civil Engineering, College of Engineering, National Taiwan University, No. 1, Sec. 4, Roosevelt Road, Taipei 10617, Taiwan; d07521014@ntu.edu.tw (D.-B.N.); r06521237@ntu.edu.tw (W.-S.L.)

**Keywords:** long-term, creep, shrinkage, strain, stress, concrete filled steel tube, model

## Abstract

A concrete-filled steel tube (CFT) combines the advantages of concrete and steel in construction and structural applications. However, research on the time-dependent deformation of the CFT under long-term sustained loading are still limited, particularly for stress transfer between the steel tube and concrete due to creep. This study investigated the creep behavior of CFT over a long period of 400 days. The creep and shrinkage strain of CFT was significantly lower than those of concrete that was not confined within a steel tube. The vertical strains of the steel tube and concrete core were almost identical, and it was shown that they were well bonded and acted as a composite. The vertical stress of steel increased by 32.7%, whereas the vertical stress of concrete decreased by 15.8% at 375 days. The stress transfer is notable and cannot be neglected in CFT design. Moreover, the results of creep and shrinkage were compared to prediction values of the B4 model and B4-TW model to verify their validity.

## 1. Introduction

Concrete-filled steel tubes are composite structural members made of a hollow steel tube filled with concrete. These composites have been widely used in practical fields because of their high ductility, high strength, good fireproof properties, excellent seismic resistance, low creep and shrinkage benefits in comparison with reinforced concrete or steel structures [1,2,3]. Nevertheless, one of disadvantage of CFT for building applications is time-dependent deformation due to creep and the shrinkage strain of the concrete core. These two inter-related phenomena can lead to a gap between the steel tube and the concrete and affect the strength and stability of composite member.

Most experiments have analyzed the time-dependent behavior of the CFT with various affecting parameters, including structure shapes, types of concrete and steel tubes, and applied stress. The creep and shrinkage of square and rectangle section specimens were tested by Uy et al. [4] and Han et al. [5], respectively. The circular cross section generally provides better confinement compared to the rectangular sections, which has been shown in a number of studies. Y. Wang et al. [6] and Ma et al. [7], determined the creep behavior of concrete-filled steel tubes using normal strength concrete and high strength concrete, respectively. They found the creep strain of CFT specimens to be significantly lower than that for unconfined concrete. Similar dimensions of specimens were tested by Ma [7] and D. J. Zhang et al. [8], who measured the creep and stress–strain relationships of the CFT. R. Zhang et al. [9] studied the creep behavior of CFT with various contents of expansive agent and indicated that the creep coefficient of the concrete structure increases with a reduction in the amount of expansive additives. Lehman et al. [10] compared the strain of CFT using self-consolidating concrete and supplementary cementitious materials concrete. The results showed that sealed cylinders of supplementary cementitious materials and CFT specimens sustained lower creep strains than the comparable self-consolidating concrete specimens. Moreover, Lai et al. [11] investigated the shrinkage strain of CFT specimens based on changing the size of the steel tube, compressive strength and amount of fly ash.

Additionally, some researchers investigated the strain and stress of the steel tube and concrete core of the CFT member. Ichinose et al. [12] and Y. Wang et al. [13] determined the strain of the concrete core and steel tube due to creep and shrinkage with different locations of gauges. Ichinose carried out experiments in which the humidity and temperatures were constant, whereas Wang tested in an ambient environment. The results showed differing strain data between the steel tube and concrete core. Kwon et al. [14] indicated that the strains within CFT components have the same magnitude over time, while vertical stress in the steel tube increases with time and the vertical stress of the concrete decreases over time. Wang et al. [15] showed the effects of different load ratios on the stress of the concrete core and steel tube, with the higher sustained loading level ranging between 40% and 80% of the strength of the specimens.

The CFT is made from two materials so that the composite has different properties and the time-dependent deformation is also more complex than separated components. Although some researchers have performed analyses of the properties of long-term behaviors, the time-dependent deformation of CFT under long-term loading has not been fully studied. Notably, a few studies investigated the stress transfer between the steel tube and concrete due to creep.

## 2. Research Significance

Long term creep tests on the time-dependent deformation behavior of CFT using high strength self-compacting concrete (SCC) embedded with a vibrating wire strain gauge (VWSG) were conducted for 400 days. The observation and estimation of the stress transfer between the concrete core and steel tube under a sustained load were also provided. The stress transfer magnitude cannot be neglected and must be taken into consideration in CFT design. Moreover, the measured creep and shrinkage values were compared to prediction values of the B4 model and B4-TW model to verify their validity.

## 3. Experimental Program

### 3.1. Materials and Mix Proportion

The cementitious materials used in the study were cement, slag and silica fume. Type II Portland cement complying with ASTM C150 [16] was used with a fineness of 347 m^2^/kg. Mineral admixtures included ground-granulated blast-furnace slag (GGBS) and silica fume (SF) with a specific surface area of 435 m^2^/kg and 15,000 m^2^/kg, respectively. The used aggregates were fine, with fineness modulus of 3.1 and sandstone was used as a coarse aggregate (CA) with a nominal maximum size of 12 mm. To enhance the workability, a high-range water-reducing agent (type F) was used as the superplasticizer (SP) admixture. An expansive additive (EA) was incorporated into all specimens to reduce the shrinkage. The mix proportion of the concrete is presented in Table 1. The concrete had a ratio of sand to aggregates (s/a) of 0.48 and water–cementitious material ratio (w/cm) of 0.24. The amount of SP and EA was 14.3 kg/cm^3^ (2% by weight of cementitious material), 20kg/cm^3^ (approximately 3% by weight of cementitious material), respectively.

### 3.2. Specimens Design

A total of six CFTs used cylindrical steel tubes with an in outer diameter of 108 mm, height of 300 mm and steel tube thickness of 3 mm. The steel tube had a yield strength of 250 MPa, grade satisfied ASTM A36. The CFT specimens had a diameter to thickness ratio (D/t) of 36, and a steel ratio (proportion of steel area to concrete area) of 12.1%. The composite members were not only carried out on basic creep and autogenous shrinkage tests but also used to determine the strain and stress behavior of the steel tube and concrete core. The CFT specimens were only used for basic creep and autogenous shrinkage tests because the role of the steel tube is to act as a permanent framework to maintain an enclosed environment so that concrete core in the steel tube is considered as a sealed specimen. The creep tests of the SCC specimens were conducted on Φ100 mm × 300 mm cylinders, while the shrinkage and length change tests of SCC specimens used two different sizes: Φ100 mm × 300 mm cylinders and 100 mm × 100 mm × 285 mm prisms. Each test set included three specimens. The specimens used in creep, shrinkage and length change tests are presented in Table 2.

The compressive strength and elastic modulus tests were conducted with cylindrical specimens of 100 mm by 200 mm dimensions. A total of 90 specimens were prepared to determine the mechanical properties at different times. Sixty specimens were used to investigate the strength and modulus of elasticity for ages of concrete at 7, 14, 28, 56 and 91 days. Another thirty cylindrical specimens were only evaluated for compressive strength in five creep tests at loading ages of 14 days and 28 days.

### 3.3. Workability Test

All test specimens were prepared in the laboratory and cured in controlled conditions. The slump test of the SCC mixture was conducted by Abrams cone, according to ASTM C143 [17], while the slump flow diameter and T50 spread time were calculated as ASTM C1611 [18]. Furthermore, to determine the “time-zero” for autogenous shrinkage and total shrinkage measurements, the initial and final setting time of the concrete were evaluated by penetration resistance according to ASTM C403 [19].

### 3.4. Compressive Strength and Elastic Modulus Tests

The compressive strength and the modulus of elasticity were tested according to ASTM C39 [20] and ASTM C469 [21], respectively. The SCC mixture was poured into the cylindrical mold. After completion of the casting, the top surface was covered with a plastic sheet to prevent water evaporation. The specimens were de-molded after 24 h and cured in standardized conditions (temperature of 23 ± 2 °C, relative humidity of 95% above) until the desired age for testing. Before the specimen was placed on the compressive machine, the surfaces of specimens were smoothed by a grinder. The compression tests were conducted using a material testing system MTS 810 platform. Linear variable displacement transducers (LVDT) were installed on the periphery of the specimen to determine the modulus of elasticity. The sixty specimens were tested for both the compressive strength and the modulus of elasticity at different time points. Lastly, thirty cylindrical specimens were cured in the same conditions as creep specimens for determination of their compressive strengths at the designated time.

### 3.5. Creep Tests

**Figure 1:**
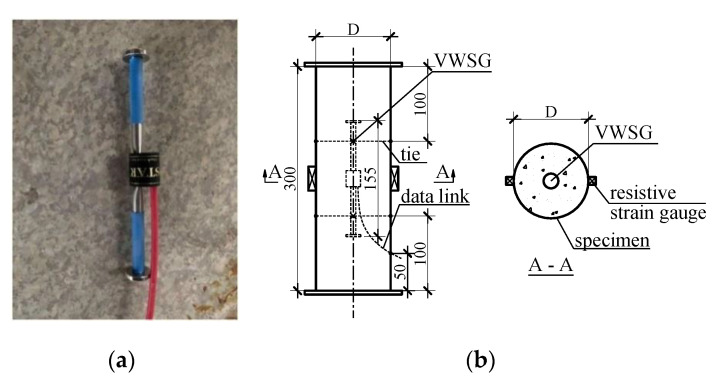
Strain gauges embedded in cylindrical specimen: (**a**) vibrating wire strain gauge (VWSG), (**b**) positions of strain gauges in specimen.

The creep tests of the CFT and SCC specimens were conducted according to ASTM C512 [22]. All time-dependent deformations of cylindrical specimens were measured with the embedded VWSG that were installed in the center of the mold. The strain gauge was made from stainless steel and had a length of 155 mm and a diameter of 19 mm, as shown in Figure 1a. These gauges were linked to the computer and the vertical strain and temperature of the concrete could be measured automatically by data acquisition software. In addition to the VWSG, the CFT specimens had two other resistive strain gauges installed outside of the specimens for recording the elastic strain of the steel tube, as illustrated in Figure 1b. In fact, the temperature changes of the specimens and the environment during the experiment can have undesirable effects on the measured data. However, in this study the VWSG with temperature compensation function can calculate exactly the strain of the specimens by the following equation

(1) $$\zeta =\left(R_{1}-R_{0}\right)K+\left(T_{1}-T_{0}\right)\left(C_{1}-C_{2}\right)$$

where *ζ* is temperature-compensated strain; $R_{1}$ and $R_{0}$ are current and initial strain (10^−6^ mm/mm), respectively; *K* is coefficient of strain gauge, which is 0.98; $T_{1}$ and $T_{0}$ are current and initial temperature of specimen (°C), respectively; $C_{1}$ is thermal-expansion coefficient of steel, $C_{1}=12.2\;\left(10^{-6}/{}^{{}^{\circ}}C\right)$; $C_{0}$ is thermal-expansion coefficient of concrete, $C_{0}=10.4\;\left(10^{-6}/{}^{{}^{\circ}}C\right)$.

**Figure 2:**
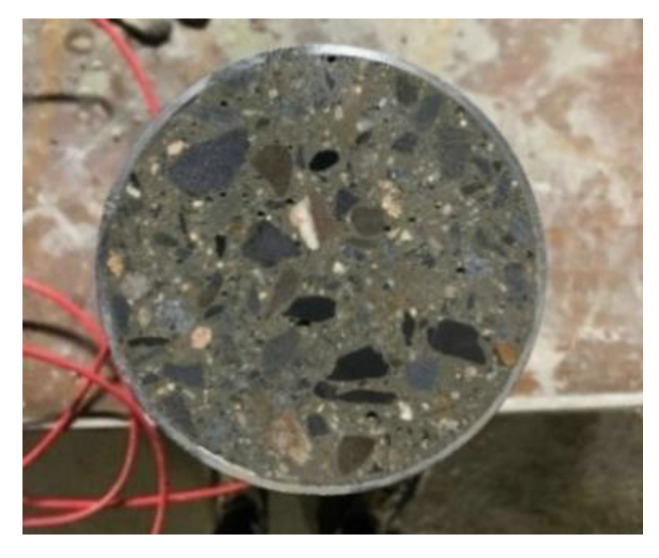
Cross section of CFT specimen.

**Figure 3:**
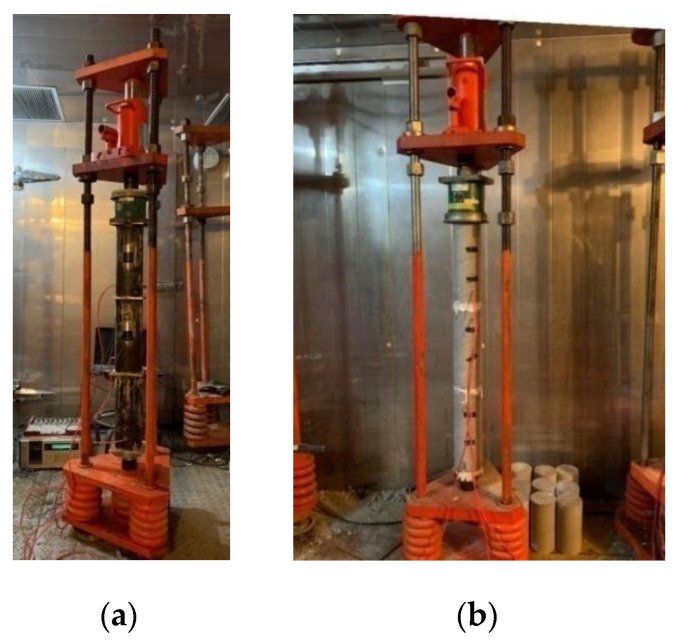
Setup for creep test with loading frame: (**a**) CFT specimens, (**b**) SCC specimens.

The bottom end of each steel tube of the CFT specimens was welded to a steel base plate of 13 cm × 13 cm in cross section. After the VWSG was installed, the concrete was filled into the mold, and immediately after casting, a plastic sheet was coated on the exposed surface to prevent evaporation. At loading ages of 14 days and 28 days from the casting, the specimens were grinded smooth, as shown in Figure 2, and another steel plate was welded on the top of steel tube. The creep of CFT specimens was applied as sustained stress by the loading frames, as presented in Figure 3a. Each of the frames could test three specimens at once.

While the CFT specimens were only subjected to a basic creep test, for the SCC specimens both basic creep and total creep were measured. The VWSG was also preinstalled in the mold of the SCC specimens and then the concrete was poured in. After 24 h from the casting, all the specimens were demolded and placed in moist curing conditions until the loading ages of 14 days and 28 days. After curing, the specimens of the total creep experiment were moved to be stored in a drying room with an automatically controlled temperature of 23 ± 2 °C and relative humidity of 50 ± 2%, whereas the basic creep specimens were s = kept in the moist environment (temperature of 23 ± 2 °C, relative humidity of 95% or above) until the end of test. The SCC specimens were connected by high strength gypsum before being put into the loading frame as presented in Figure 3b. The quality of the joints was usually checked with a spirit-level until the applied stress on specimens was stable. The value of stress was calculated exactly based on the compressive strength of the cylindrical specimens which were cured in the same conditions as the creep tests. The specimens were kept under sustained loading over a year.

### 3.6. Shrinkage Tests

**Figure 4:**
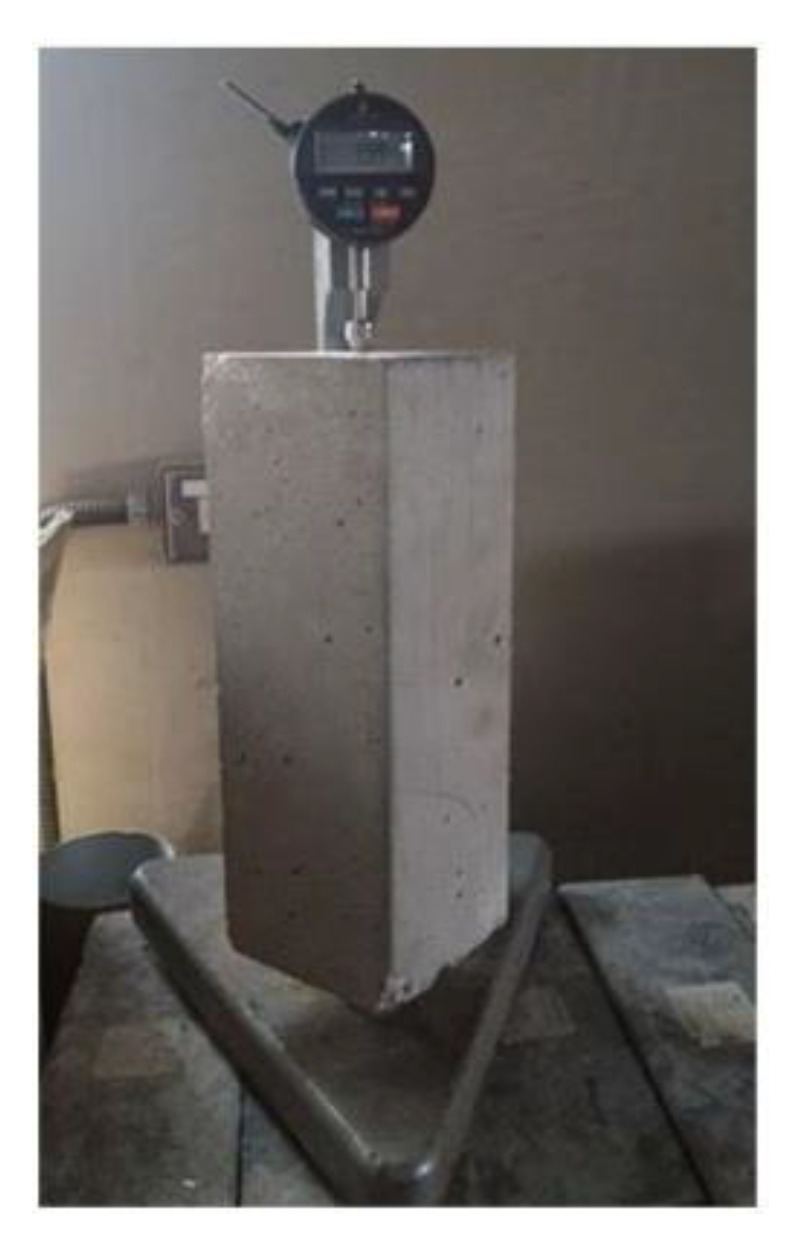
Total shrinkage of the prismatic specimen measured by length comparator.

The concrete core of the CFT specimens only experiences autogenous shrinkage as a result of the self-desiccation process, but the SCC specimens were assessed both in terms of total shrinkage and length change up to 400 days. All shrinkage specimens were demolded 24 h after casting and cured in a standardized chamber (temperature of 23 ± 2 °C, relative humidity of 95% or above). When the concrete ages were cured for 14 days and 28 days, the C-SCC-TS14 and P-SCC-TS28 specimens—for which total shrinkage was determined—were stored in a drying room (temperature of 23 ± 2 °C, relative humidity of 50 ± 2%), respectively. Moreover, for the purpose of evaluation, the length change effects of the concrete on the creep data, the C-SCC-LW14 and P-SCC-LW28 specimens were placed in the same environment as the basic creep specimens at a designated time. The shrinkage strain of the cylindrical specimens can be recorded at an initial setting time (the beginning of solidification) by the VWSG. After the initial setting time of fresh concrete was determined based on ASTM C403 [19], the shrinkage data were collected by the VWSG embedded inside the specimens, the same as described in the creep tests. However, the shrinkage and length change of 100 mm × 100 mm × 285 mm prism specimens were only measured manually at 28 days after curing in standard conditions by the length comparator, according to ASTM C157 [23], as shown in Figure 4. The value of the prisms is for the purpose of comparisons with the data of the method using VSWG.

## 4. Prediction Models for Creep and Shrinkage

Many present models predict the creep and shrinkage of concrete, for example the ACI 209R-92 [24,25], fib mode code 2010 [26], B3 model [25,27], B4 model [28], B4-TW model [29,30], GL2000 [25,31,32], and JSCE model [33]. However, no prediction models consider the effect of cement replacement with mineral admixtures on time-dependent deformation, except for the B4-TW model and the B4 model. The B4-TW model was developed based on the B4 model, they have same main creep and shrinkage formulae but their details are different.

### 4.1. Creep Prediction Models

#### 4.1.1. Creep of B4-TW Model

According to the B4-TW model, the creep compliance function is given by

(2) $$J\left(\hat{t},{\hat{t}}^{\prime }\right)=q_{1}+C_{b}\left(\hat{t},{\hat{t}}^{\prime }\right)+C_{d}\left(\hat{t},{\hat{t}}^{\prime },{\tilde{t}}_{0}\right)$$

where $\hat{t}$, ${\hat{t}}^{\prime }$, ${\tilde{t}}_{0}$ are temperature corrected current age, age at loading and age at exposure, respectively (days); $C_{b}\left(\hat{t},{\hat{t}}^{\prime }\right)$ is the basic creep compliance; $C_{d}\left(\hat{t},{\hat{t}}^{\prime },{\tilde{t}}_{0}\right)$ is the drying creep compliance.

(3) $$C_{b}\left(\hat{t},{\hat{t}}^{\prime }\right)=q_{2}Q\left(\hat{t},{\hat{t}}^{\prime }\right)+q_{3}\ln\left[1+{\left(\hat{t}-{\hat{t}}^{\prime }\right)}^{n}\right]+q_{4}\ln\left(\frac{\hat{t}}{{\hat{t}}^{\prime }}\right)$$

In which $Q\left(\hat{t},{\hat{t}}^{\prime }\right)$ is the function calculated by numerical evaluation of the integral; *n* is the empirical parameter, (*n* = 1 for normal concrete).

(4) $$C_{d}\left(\hat{t},{\hat{t}}^{\prime },{\tilde{t}}_{0}\right)=q_{5}{\left\{\exp\left[-p_{5H}H\left(\hat{t},{\tilde{t}}_{0}\right)\right]-\exp\left[-p_{5H}H_{c}\left({{\hat{t}}^{\prime }}_{0},{\tilde{t}}_{0}\right)\right]\right\}}^{0.5}$$

With ${{\hat{t}}^{\prime }}_{0}=\max\left({\hat{t}}^{\prime },{\tilde{t}}_{0}\right)\;if\;\hat{t}\geq {{\hat{t}}^{\prime }}_{0}$, where $H\left(\hat{t},{\tilde{t}}_{0}\right)$ and $H_{c}\left({{\hat{t}}^{\prime }}_{0},{\tilde{t}}_{0}\right)$ are spatial averages of pore relative humidity; $q_{i}$ is the compliance parameter.

(5) $$q_{1}=\frac{p_{1}}{E_{cm28}}=\frac{p_{1}}{3831\sqrt{f_{cm28}}}$$

(6) $$q_{2}=\frac{p_{2}}{1\;GPa}{\left(\frac{w/cm}{0.38}\right)}^{p_{2w}}{\left(\frac{6.5cm}{\rho }\right)}^{p_{2c}}$$

(7) $$q_{3}=p_{3}q_{2}{\left(\frac{a/cm}{6}\right)}^{p_{3a}}{\left(\frac{w/cm}{0.38}\right)}^{p_{3w}}$$

(8) $$q_{4}=\frac{p_{4}}{1\;GPa}{\left(\frac{a/cm}{6}\right)}^{p_{4a}}{\left(\frac{w/cm}{0.38}\right)}^{p_{4w}}$$

(9) $$q_{5}=\frac{p_{5}}{1\;GPa}{\left(\frac{a/cm}{6}\right)}^{p_{5a}}{\left(\frac{w/cm}{0.38}\right)}^{p_{5w}}{\left|k_{h}\varepsilon_{sh\infty }\left({\tilde{t}}_{0}\right)\right|}^{p_{5\varepsilon }}$$

where $E_{cm28}$ is the modulus of elasticity at 28 days; $f_{cm28}$ is the compressive strength at 28 days; a, cm, and w present aggregate, cementitious material and water content (kg/m^3^), respectively; ρ is the concrete density (kg/m^3^); factors $p_{i}$, $p_{5H}$ and exponents $p_{2w}$, $p_{2c}$, $p_{3a}$, $p_{3w}$, $p_{4a}$, $p_{4w}$, $p_{5a}$, $p_{5w}$, $p_{5\varepsilon }$ depend on the cement type; $k_{h}$ is the humidity dependence factor; $\varepsilon_{sh\infty }\left({\tilde{t}}_{0}\right)$ is the ultimate shrinkage.

#### 4.1.2. Creep of B4 Model

The B4 model has different equation details in comparison with the B4-TW model. The creep compliance function is determined by

(10) $$J\left(\hat{t},{\hat{t}}^{\prime }\right)=q_{1}+R_{T}C_{b}\left(\hat{t},{\hat{t}}^{\prime }\right)+C_{d}\left(\hat{t},{\hat{t}}^{\prime },{\tilde{t}}_{0}\right)$$

(11) $$q_{1}=\frac{p_{1}}{E_{cm28}}=\frac{p_{1}}{4734\sqrt{f_{cm28}}}$$

(12) $$q_{2}=\frac{p_{2}}{1\;GPa}{\left(\frac{w/c}{0.38}\right)}^{p_{2w}}$$

where $R_{T}$ is an acceleration of time factor; c is the cement content (kg/m^3^); $C_{b}\left(\hat{t},{\hat{t}}^{\prime }\right)$ is the basic creep compliance, as given in Equation (3); $C_{d}\left(\hat{t},{\hat{t}}^{\prime },{\tilde{t}}_{0}\right)$ is the drying creep compliance, as given in Equation (4).

The $q_{3}$, $q_{4}$, $q_{5}$ factors in the B4 model are almost the same as the B4-TW model, except the content of cementitious material (*cm*) is replaced by the cement content (*c*).

### 4.2. Shrinkage Prediction Models

#### 4.2.1. Shrinkage of B4-TW Model

According to the B4-TW model, the total shrinkage strain is given as

(13) $$\varepsilon_{cs}\left(\tilde{t},{\tilde{t}}_{0}\right)=\varepsilon_{sh}\left(\tilde{t},{\tilde{t}}_{0}\right)+\varepsilon_{au}\left(\tilde{t},{\tilde{t}}_{0}\right)-\varepsilon_{au}\left({\tilde{t}}_{0}\right)$$

where $\tilde{t}$ is the temperature corrected exposure duration (days); $\varepsilon_{sh}\left(\tilde{t},{\tilde{t}}_{0}\right)$ is the drying shrinkage strain; $\varepsilon_{au}\left(\tilde{t},{\tilde{t}}_{0}\right)$, $\varepsilon_{au}\left({\tilde{t}}_{0}\right)$ are autogenous shrinkage strain at $\tilde{t}$ days and ${\tilde{t}}_{0}$ days, respectively.

(14) $$\varepsilon_{sh}\left(\tilde{t},{\tilde{t}}_{0}\right)=f_{sh,cem}\varepsilon_{sh\infty }\left({\tilde{t}}_{0}\right)k_{h}\tanh\sqrt{\frac{\tilde{t}}{f_{sh,\tau }\tau_{sh}}}$$

(15) $$\varepsilon_{sh\infty }\left({\tilde{t}}_{0}\right)=-\varepsilon_{0}k_{\varepsilon \alpha }\frac{E\left(7\beta_{Th}+600\beta_{Ts}\right)}{E\left({\tilde{t}}_{0}+\tau_{sh}\beta_{Ts}\right)}$$

(16) $$\tau_{sh}=\tau_{0}k_{\tau a}{\left(2k_{s}V/S\right)}^{2}$$

(17) $$\varepsilon_{0}=\varepsilon_{cem}{\left(\frac{a/cm}{6}\right)}^{p_{\varepsilon a}}{\left(\frac{w/cm}{0.38}\right)}^{p_{\varepsilon w}}{\left(\frac{6.5cm}{\rho }\right)}^{p_{\varepsilon c}}$$

(18) $$\tau_{0}=\tau_{cem}{\left(\frac{a/cm}{6}\right)}^{p_{\varepsilon a}}{\left(\frac{w/cm}{0.38}\right)}^{p_{\varepsilon w}}{\left(\frac{6.5cm}{\rho }\right)}^{p_{\varepsilon c}}$$

In which $k_{\tau a}$, $k_{\varepsilon a}$ are the factors depending on the type of aggregate; $k_{s}$ is cross-section shape correction factor; $\frac{E\left(7\beta_{Th}+600\beta_{Ts}\right)}{E\left({\tilde{t}}_{0}+\tau_{sh}\beta_{Ts}\right)}$ is the factor to account for the time dependence of ultimate shrinkage; *V*/*S* is the volume to surface ratio.

The autogenous shrinkage of concrete without mineral admixture is expressed as

(19) $$\varepsilon_{au}\left(\tilde{t},{\tilde{t}}_{0}\right)=\varepsilon_{au\infty }{\left[1+{\left(\frac{\tau_{au}}{\tilde{t}+{\tilde{t}}_{0}}\right)}^{\alpha }\right]}^{r_{t}}$$

(20) $$\varepsilon_{au\infty }=-\varepsilon_{au,cem}{\left(\frac{a/cm}{6}\right)}^{r_{\varepsilon a}}{\left(\frac{w/cm}{0.38}\right)}^{r_{\varepsilon w}}$$

(21) $$\tau_{au}=\tau_{au,cem}{\left(\frac{w/cm}{0.38}\right)}^{r_{\tau w}}$$

(22) $$\alpha =r_{\alpha }\left(\frac{w/cm}{0.38}\right)$$

Autogenous shrinkage of concrete with mineral admixtures is calculated by

(23) $$\varepsilon_{au}\left(\tilde{t},{\tilde{t}}_{0}\right)=\varepsilon_{au\infty }\left\{{}_{}1-\exp\left[-0.2{\left(\tilde{t}+{\tilde{t}}_{0}\right)}^{0.5}\right]\right\}$$

(24) $$\varepsilon_{au\infty }=-f_{au,cem}\varepsilon_{au,cem}{\left(\frac{a/cm}{6}\right)}^{r_{\varepsilon a}}{\left(\frac{w/cm}{0.38}\right)}^{f_{au,wc}r_{\varepsilon w}}$$

where the parameters $\varepsilon_{cem}$, $\tau_{cem}$, $\tau_{au,cem}$, $\varepsilon_{au,cem}$, and the exponents $p_{\varepsilon a}$, $p_{\varepsilon w}$, $p_{\varepsilon c}$, $p_{\tau a}$, $p_{\tau w}$, $p_{\tau c}$, $r_{\tau w}$, $r_{t}$, $r_{\alpha }$, $r_{\varepsilon a}$, $r_{\varepsilon w}$ are the cement type dependent; $f_{sh,cem}$, $f_{sh,\tau }$, $f_{au,cem}$, $f_{au,wc}$ are shrinkage parameters.

#### 4.2.2. Shrinkage of B4 Model

The B4 model includes total shrinkage, expressed by

(25) $$\varepsilon_{cs}\left(\tilde{t},{\tilde{t}}_{0}\right)=\varepsilon_{sh}\left(\tilde{t},{\tilde{t}}_{0}\right)+\varepsilon_{au}\left(\tilde{t},{\tilde{t}}_{0}\right)$$

(26) $$\varepsilon_{sh}\left(\tilde{t},{\tilde{t}}_{0}\right)=\varepsilon_{sh\infty }\left({\tilde{t}}_{0}\right)k_{h}\tanh\sqrt{\frac{\tilde{t}}{\tau_{sh}}}$$

Other shrinkage formulae of the B4 model are the same as in the case of the concrete without the mineral admixture of the B4-TW model, only the cementitious material content of factors $\varepsilon_{0}$, $\tau_{0}$, $\varepsilon_{au\infty }$, $\tau_{au}$, α are replaced by cement content. In the case of the concrete uses mineral admixtures, the factors $p_{2}$, $p_{3}$, $p_{4}$, $p_{5}$ of creep prediction and shrinkage parameters $\tau_{cem}$, $\varepsilon_{au,cem}$, $r_{\varepsilon w}$, $r_{\alpha }$ are adjusted as in the theory of the B4 model [28].

## 5. Results and Discussion

### 5.1. Workability and Mechanical Properties

The SCC concrete had a slump flow value of 570 mm, and T_50_ spread time of 16 s. The initial setting time and final setting time of the concrete were 539 min and 729 min, respectively.

The average compressive strength and modulus of elasticity of SCC were tested on 12 specimens for each age at 7, 14, 28, 56 and 91 days, as shown in Table 3. The results indicated that the 14-day compressive strength could reach 88% of the 28-day strength. The compressive strength of the SCC was 86 MPa at 28 days, so it was classified as a high strength concrete [34] and this value was also used for calculation of the prediction models. The average compressive strength development was gradually increased to 92 MPa at 91 days. The modulus of elasticity was 32.8 GPa at 28 days and, then the value tended to stabilize.

### 5.2. Stress Conversion

**Figure 5:**
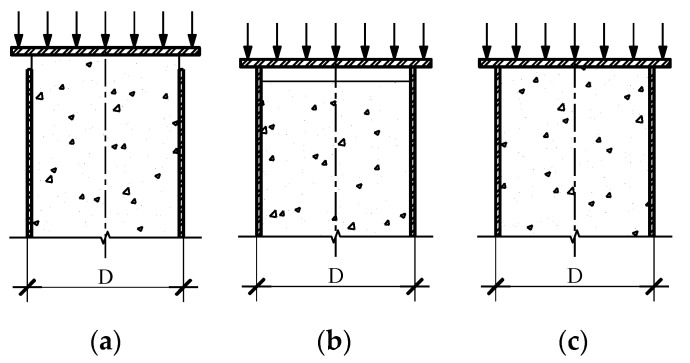
Applied stress on plane section of CFT: (**a**) stress on concrete core, (**b**) stress on steel tube, (**c**) stress on composite member.

The CFT combines concrete and steel tubes so that the stress that impacts on these specimens is not the same as plain concrete. It is well known that creep and shrinkage of the concrete core directly affect the time-dependent deformation of CFT. If the surfaces of the components are not the same level, as presented in Figure 5a,b, the steel tube and concrete core work as separate constituents and the applied stress on the specimens cannot be calculated correctly. In case of the plane remains plane, as shown in Figure 5c, the stress and strain are uniform throughout the steel tube and concrete core so that two components of CFT can be designed as parts of a single member and full composite function can work accurately.

According to the theory of elasticity, the stress of CFT is converted to find exactly applied loading values on the specimens. The theory load of CFT can be expressed as

(27) $$N_{cft}=N_{cft,s}+N_{cft,c}$$

where $N_{cft}$, $N_{cft,c}$, $N_{cft,s}$ are the axial load of CFT specimen, concrete and steel component, respectively (kN).

It is assumed that the plane remains plane under axial compression, which is the essence of strain compatibility so that the steel strain is the same as the concrete strain at all locations and can be calculated by the following equation

(28) $$\varepsilon_{cft,s}=\varepsilon_{cft,c}=\frac{N_{cft,c}}{A_{cft,c}\cdot E_{c}}$$

The load applies to the steel tube can be calculated on that of the concrete core as

(29) $$N_{cft,s}=\sigma_{cft,s}\cdot A_{cft,s}=\left(E_{s}\cdot \varepsilon_{cft,s}\right)A_{cft,s}$$

In which $A_{cft,c}$, $A_{cft,s}$ are cross sections of the concrete core and steel tube, respectively (mm^2^); $E_{c}$, $E_{s}$ are the elastic modulus of concrete core and steel tube, respectively (MPa); $\varepsilon_{cft,c}$, $\varepsilon_{cft,s}$ are the elastic strain of concrete core and steel tube, respectively.

The loading force of the concrete core of the CFT can be calculated the same as with the SCC specimens.

(30) $$N_{cft,c}=N_{c}=n_{c}\cdot {f^{\prime }}_{c}\left(t^{\prime }\right)\cdot A_{c}$$

where $N_{c}$ is the axial load (kN); $n_{c}$ is the designated stress ratio; ${f^{\prime }}_{c}\left(t^{\prime }\right)$ is the compressive strength at loading age *t*′ (MPa); $A_{c}$ is the cross section of the concrete component (mm^2^).

**Figure 6:**
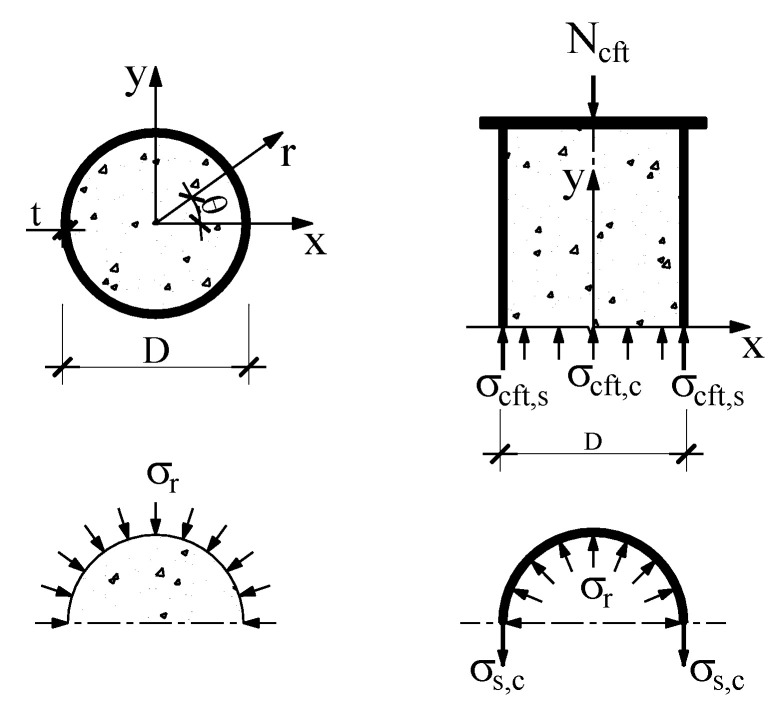
Different stresses in concrete filled steel tube.

When CFT is under axial loading, various cylinder stresses appear in the composite member, including vertical stress of the steel tube ($\sigma_{cft,s}$ or $\sigma_{s,v}$) and vertical stress of the concrete core ($\sigma_{cft,c}$), circumferential stress in steel tube ($\sigma_{s,c}$), radial stress ($\sigma_{r}$) as shown in Figure 6.

The stress that occurs on loading of the composite specimen was found to be directly proportional to the strain. The stress–strain relationship of the CFT subjected to axial compression follows Hook’s law within the elastic range.

(31) $$\left[\begin{array}{l} \sigma_{s,v}\\ \sigma_{s,c} \end{array}\right]=\frac{E_{s}}{1-\upsilon_{s}^{2}}\left[\begin{array}{l} \;1\;\upsilon_{s}\\ \upsilon_{s}\;1 \end{array}\right]\left[\begin{array}{l} \varepsilon_{s,v}\\ \varepsilon_{s,c} \end{array}\right]$$

The vertical stress of steel tube $\sigma_{cft,s}$ and concrete core $\sigma_{cft,c}$ can be calculated by

(32) $$\sigma_{cft,s}=\sigma_{s,v}=\frac{E_{s}}{1-\upsilon_{s}^{2}}\left(\varepsilon_{s,v}+\upsilon_{s}\varepsilon_{s,c}\right)$$

(33) $$\sigma_{cft,c}=\frac{N_{cft,c}}{A_{e,cft,c}}=\frac{N_{cft}-N_{cft,s}}{A_{e,cft,c}}$$

In which $\sigma_{s,v}$, $\sigma_{s,c}$ are the vertical and circumferential stress in steel tube (MPa), respectively; $\varepsilon_{s,v}$, $\varepsilon_{s,c}$ are the vertical and circumferential strain of the steel tube (μ*ε*); $\upsilon_{s}$ is the Poisson’s ratio of steel ($\upsilon_{s}=-\varepsilon_{s,c}/\varepsilon_{s,v}$); $A_{e,cft,c}\left(t^{\prime }\right)$ is the conversion area of the concrete core (mm^2^).

Additionally, when the specimens are embedded with the VWSG inside the specimen, the conversion area can be obtained by

(34) $$A_{e,c}\left(t^{\prime }\right)=n\left(t^{\prime }\right)\cdot A_{vw}+A_{c}$$

(35) $$n\left(t^{\prime }\right)=\frac{E_{vw}}{E_{mts}\left(t\prime \right)}$$

where $A_{vw}$ is the cross section of the VWSG (mm^2^); $n\left(t^{\prime }\right)$ is the conversion factor; $E_{vw}$ is the modulus of elasticity of the VWSG (MPa); $E_{mts}\left(t^{\prime }\right)$ is the elastic modulus of the test specimens at loading age *t*′.

The conversion stress $\sigma_{c}\left(t^{\prime }\right)$ and real stress ratio $n_{c}\left(t^{\prime }\right)$ are determined by the following equations

(36) $$\sigma_{c}\left(t^{\prime }\right)=\frac{N_{c}\left(t^{\prime }\right)}{A_{e,c}\left(t^{\prime }\right)}$$

(37) $$n_{c}\left(t^{\prime }\right)=\frac{\sigma_{c}\left(t^{\prime }\right)}{f_{c}\left(t^{\prime }\right)}$$

According to the loading force equations with the designated stress ratio ($n_{c}$) of 0.3, the load value of SCC specimens is 180 kN and 202 kN at 14 days and 28 days, respectively, and the axial load of the CFT specimens ($N_{cft}$) is 325 kN at 14 days. The elastic strain of the concrete core and steel tube can be determined by the strain gauges. The elastic modulus of the steel tube and stainless steel of the VWSG are 200 GPa and 195 GPa, respectively. Poisson’s ratio of the steel $\upsilon_{s}$ is 0.3. The conversion of the designated stress ratios to real stress ratios by consideration of the VWSG embedded in the specimen is given in Table 4.

### 5.3. Creep Behavior

#### 5.3.1. Influence of Steel Tube on Creep

**Figure 7:**
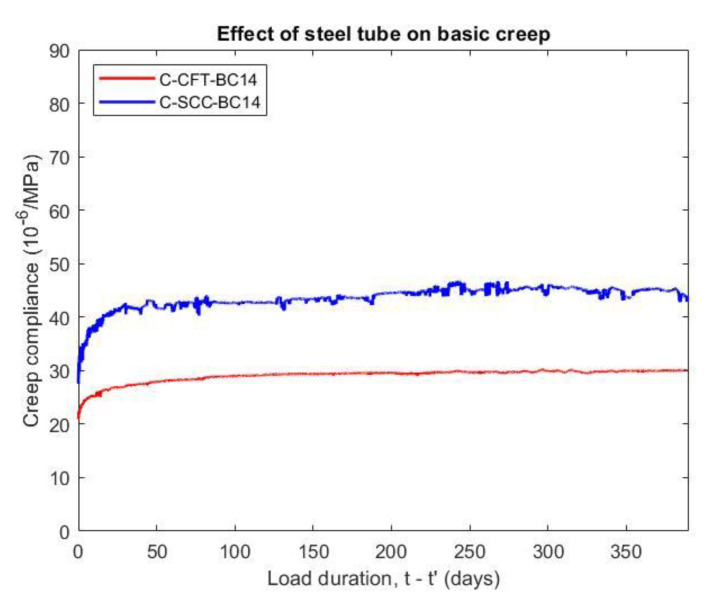
Basic creep compliance of CFT and SCC under applied stress at 14 days.

Figure 7 compares the basic creep compliance of the CFT and SCC specimens. The creep occurred from the immediate onset of loading and was larger in the first week but stabilized after. The data show that the basic creep compliance of the CFT was lower by about 33% in comparison with that of the unconfined concrete at 375 days after loading age. As the load is applied, the steel tube restrains concrete dilation and passive confinement of the steel tube significantly improves the deformation capacity of the concrete core. Although the applied stress is sustainable, the ratio of stress to strength is lower because the strength of the concrete improved by the confinement effect of the steel tube more than the plain concrete. On the other hand, the stress of the concrete component is relaxed due to possible stress transfer between the concrete and steel tube so that creep of CFT is reduced. The result for the creep of CFT herein was rather consistent with previous research. R. Zhang et al. [9] indicated that the creep compliance of the concrete specimens with lateral restraint was about 11 to 27 (10^−6^/MPa) depending on the applied loading, expansive agent content, and steel ratios.

#### 5.3.2. Influence of Loading Ages on Creep

**Figure 8:**
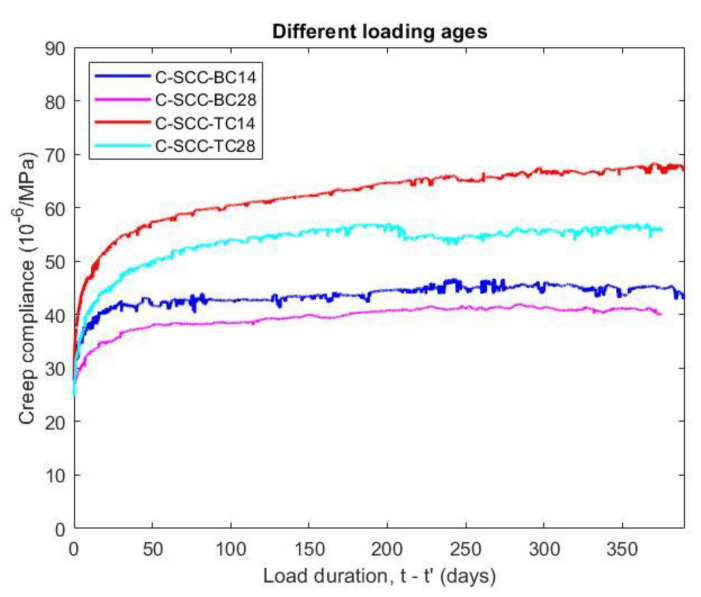
Effect of loading ages on basic and total creep compliance of SCC specimens.

The amount of creep reduces on with the loading age of the concrete, as shown in Figure 8. When the specimens were cured in moist conditions (relative humidity of 95% or above), the effect of the loading age on the basic creep is quite small. The basic creep compliance of the loading age at 14 days was 12.5% higher than that of the loading age at 28 days after applied stress for 375 days.

When the specimens were stored in the dry environment (relative humidity of 50 ± 2%), the loading age significantly affected the creep values. Figure 8 illustrates that the total creep compliance of specimens with a loading age of 14 days and 28 days were different up to 19.8% at 375 days after loading. Although the ratio of stress to compressive strength is constant, an earlier increase in stress accompanies a higher creep. The reason for this is the lower compressive strength at the early stage corresponding to a larger applied stress to strength ratio.

#### 5.3.3. Influence of Relative Humidity on Creep

The total creep compliance was 49% and 40% higher than the basic creep compliance at 375 days after loading ages of 14 days and 28 days, respectively, as observed in Figure 8. The total creep also increased at a greater rate than the basic creep at earlier periods of loading. The result demonstrates that the creep not only depends on the load and time but also on the relative humidity of the environment. While the basic creep is not influenced by the level of humidity, the total creep has a rapid drying process at an earlier age. Generally, the total creep—the sum of the basic creep and drying creep (Pickett effect)—is considerably larger than the basic creep because the amount of water reduction in the capillary pores is much faster in drying condition. Additionally, the ambient humidity effect on the moisture of the concrete, it also directly affects the cement hydration and strength of the concrete.

The elastic strain, creep strain, creep compliance and creep coefficient of the basic and total creep specimens at 375 days after loading are given in Table 5.

### 5.4. Shrinkage Behavior

#### 5.4.1. Effect of Curing Conditions on Shrinkage

**Figure 9:**
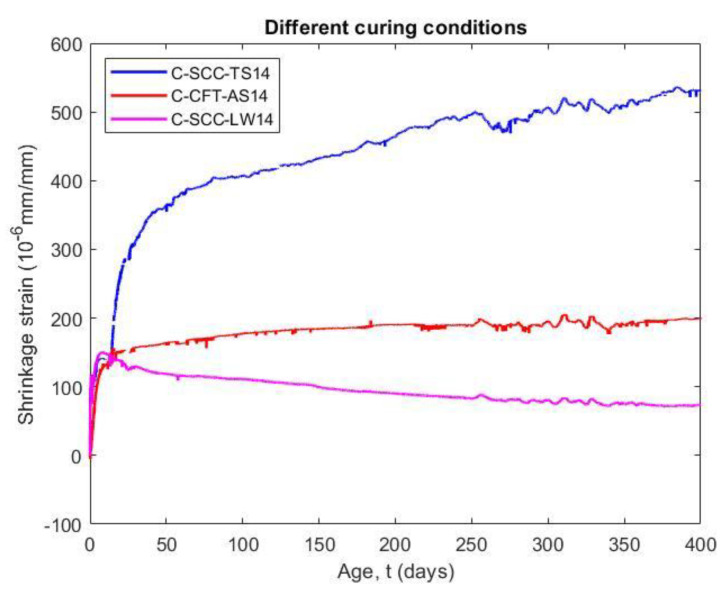
Shrinkage of CFT and SCC measured at initial setting time.

The shrinkage occurs as soon as the adsorbed water from the hydrated cement paste is removed due to the effects of cement hydration and water loss, so that the shrinkage strain was normally measured from the instant when the concrete starts to harden. The shrinkage and length change values were measured at an initial setting time, as shown in Figure 9. It can be seen that the autogenous shrinkage of the CFT as well as the total shrinkage and the length change of the plain concrete have a greater rate of shrinkage at an earlier age. The reason for this is an increase in the stiffness due to development of strength with age, known as the ageing effect. Moreover, the older concrete has a slower moisture diffusion in the exposed condition.

As a result, the autogenous shrinkage of CFT specimens (C-CFT-AS14) increased rapidly within 14 days after molding and the value reached approximately 71% of the shrinkage strain at 400 days. The shrinkage strain of the concrete stabilized from the third week. The issue that the confining effect of steel tube not only significantly restraints the shrinkage of the concrete but also prevents the swelling because of the expansive agent, is considered. Shrinkage of the CFT specimen increases only due to the result of the self-desiccation process as the macroscopic volume reduction in cementitious materials when the cement hydrates after the initial setting. The volume of hydration products is less than the sum of the volume of water and the volume of cement that is hydrated [35]. In comparison with the specimen cured in the wet condition (C-SCC-LW14), after the length change of this sample reached the highest value at 7 days, the deformation of the concrete without a steel tube trend swelled slowly by up to 0.5 times at 400 days. The main reason for the reversible shrinkage of specimens is an incorporation of additional water from the humid environment into the porous structure, as well as an increase in the water content of the adsorbed layers in the concrete structure [36]. Therefore, the length change of the concrete curing in the moist environment was 63% lower than the shrinkage of concrete core at the last day of testing (400 days).

Figure 9 also indicates that the shrinkage of the concrete core of the CFT specimens was 62% lower than the shrinkage of the plain concrete placed in a dry environment (C-SCC-TS14). The shrinkage of the concrete core significantly reduces because the concrete is cured in an enclosed moist environment by the steel tube, which has an important role in preventing the water inside of the concrete from evaporating into the environment. However, the shrinkage of the plain concrete increased because the adsorbed water in the concrete is diffused into the environment and the water held by capillary tension in small pores under 50 nm—which play an important role in drying shrinkage—moved into large capillary voids within the matrix of the hydrated cement paste or to the atmosphere [37]. For comparative purposes, Lehman et al. [10] showed that the shrinkage of self-consolidating concrete filled steel tube specimen was only 200 (10^−6^ mm/mm) at 126 days, which is equal to 44% of the SCC specimen at the same time.

When the temperature is stable—the same as in the testing condition—the total strain at age t can be calculated without thermal expansion by the following equation [38]

(38) $$\varepsilon \left(t\right)=\varepsilon_{cs}\left(t\right)+\varepsilon_{e}\left(t\right)+\varepsilon_{cr}\left(t\right)$$

**Figure 10:**
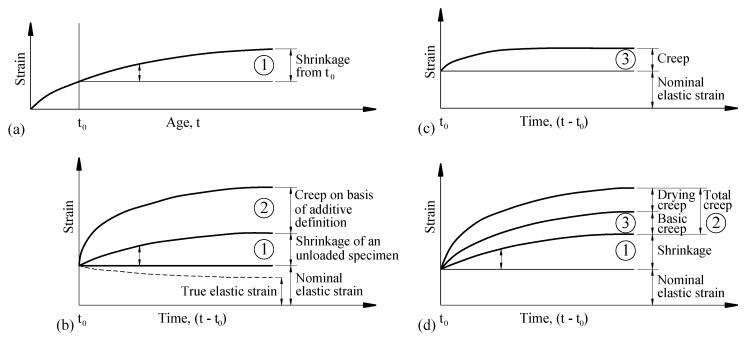
Definition of different strain of concrete: (**a**) shrinkage of an unloaded companion specimen, (**b**) change in strain of loaded and drying specimen, (**c**) creep of a loaded specimen in equilibrium with the ambient medium, (**d**) change in strain of a loaded and drying specimen [39].

The real creep strains can be obtained by subtracting the shrinkage strains from the measured creep strains, as illustrated in Figure 10 [39]. Therefore, real creep can be expressed as

(39) $$\varepsilon_{cr}\left(t\right)=\varepsilon_{cc}\left(t\right)-\varepsilon_{cs}\left(t\right)$$

In which $\varepsilon_{cs}$ is the shrinkage strain (10^−6^ mm/mm); $\varepsilon_{e}$ is the instantaneous strain (10^−6^ mm/mm), which is elastic strain if the stress is small; $\varepsilon_{cr}$ is the real creep strain (10^−6^ mm/mm), $\varepsilon_{cc}$ is the measured creep strain (10^−6^ mm/mm).

The shrinkage strain of specimens was measured after the curing of concrete in addition to the creep strain and total strain presented, and these values are presented in Table 6. The total strains of CFT and plain concrete placed in the moist conditions were 0.44 times and 0.33 times less than those of the comparable specimen in dry condition at 375 days after the curing time for 14 days, respectively.

#### 5.4.2. Effect of Curing Ages on Shrinkage

**Figure 11:**
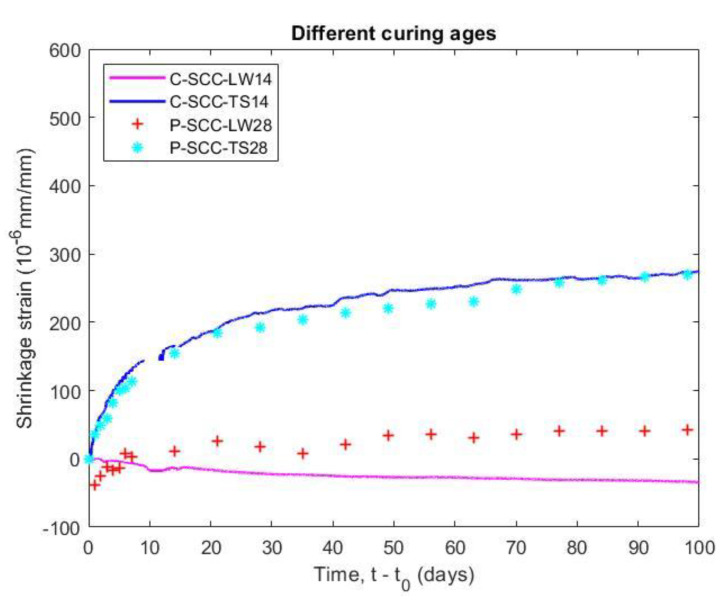
Effect of different curing ages on shrinkage of SCC specimens.

The measured time of shrinkage and length change tests were taken at 14 days and 28 days as well as loading age of creep tests, and the data are plotted in Figure 11. The result indicated that the different curing ages have a small effect on the shrinkage and length change of the SCC specimens. The total shrinkage of the prism specimen (P-SCC-TS28) had a magnitude corresponding to that of the cylindrical one (C-SCC-TS14) at 98 days after curing. The effect of the different shape on the result can be neglected in shrinkage tests when the ratio of volume to surface area (V/S) of C-SCC-TS14 and P-SCC-TS28 was 21.4 mm and 21.3 mm, respectively. Additionally, the specimens (C-SCC-LW14 and P-SCC-LW28) that were stored in the moist environment have a small length change so that the effect of shrinkage strain on basic creep value can be disregarded. The results also indicate the stabilization of moisture diffusion and micro-structure of the concrete after 14 days.

### 5.5. Strain and Stress of CFT

#### 5.5.1. Strain Behavior

**Figure 12:**
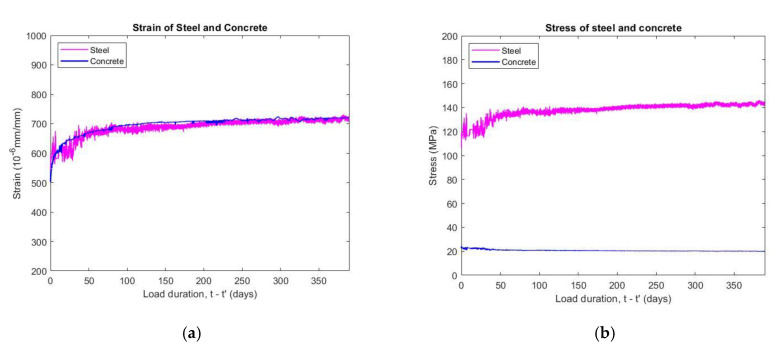
Strains and stresses of steel tube and concrete core: (**a**) vertical strains, (**b**) vertical stresses.

The strains of the CFT components were measured by separate strain gauges. As can be observed in Figure 12a, the values increase quickly at 21 days and become stable after about 50 days. The vertical strains of the steel tube and the concrete began at the time of applied loading and increased gradually up to 375 days after the applied stress. The two components of the composite member had a similar magnitude of vertical strain, with only a 1.1% difference in the strains of the steel tube and concrete core.

In the initial period after loading, the steel tube had a vertical strain value higher than the concrete core because the Poisson’s ratio of concrete is lower than that of the steel. The difference of vertical strains of CFT components also indicates that longitudinal slipping between the steel tube and concrete can occur in this stage. The vertical strain of the concrete was higher with time due to creep in addition to the larger lateral strain. However, the deformation of the concrete core is restrained by the confinement effect of the steel tube so that the vertical strain of the concrete increases equally with steel. The result confirms that the plane remains plane as well as compatibility between steel and concrete components. The test data compared suitably with those of Kwon et al. [14], who determined that vertical strains within the steel tube and concrete core have the same magnitude.

#### 5.5.2. Stress Behavior

When the CFT specimens were subjected to a sustained load of 325 kN with a conversion stress ratio of 0.32, the stress of the components can be evaluated according to the data of strain gauges. Figure 12b illustrates that the vertical stress transfer on the steel tube increases by 32.7% at 375 days after the loading age, whereas that on the concrete core drops by 15.8% of the vertical stress at the same time. The stress of composite member is influenced by strain variation, and the stresses of the CFT components are unstable due to no restraint effects on the concrete core during the initial weeks. It also can be observed clearly that the stress magnitude of fluctuation of steel tube is much large in this time. The stress transfer only occurs from a radial pressure that develops at the steel–concrete interfacial bond because the lateral expansion of the concrete core increases to a larger degree than the steel tube. The varying magnitude of stress of the steel tube also becomes smaller with time. On the other hand, the reason for the stress redistribution is that the concrete possesses creep properties but the steel does not perform creep deformation. Furthermore, the interconnection between the steel tube and the concrete core of the CFT—which has sufficient strength and stiffness to combine the steel tube and the concrete core work as a unity member—also has an important role in influencing the behavior of the composite structure.

The stress result of the composite components varied over time and was also shown by Kwon et al. [14] and Wang et al. [15]. Kwon [14] explained that the value of the vertical stress variation in the concrete core is smaller than that in the steel tube because the cross-sectional area of the concrete is much larger than the area of steel. Wang [15] indicated that the vertical stresses in the steel tubes were about 310 to 320 MPa under sustained loading between 40% and 80% of the strength, whereas vertical stress in the concrete core basically maintained a constant value of 29 to 30 MPa for specimens of 1 meter in length at 300 days. The measured stress of the CFT can also be compared to predicted value by Ya-Ju Yu [40]. In this method, the parameters used include a basic creep of the concrete (C-SCC-BC14 specimen) of 198 (10^−6^ mm/mm) at 375 days and a modulus of elasticity of 355,555 MPa, respectively, a D/t ratio of 36, and a yield strength of steel of 250 MPa, the stress transfer of the steel tube was predicted to be 7% of steel stress equal to 17.5 MPa, which is an error of 50% in comparison with the experimental data.

The vertical strains, vertical stresses, and stress transfer of CFT components varied at various days, as can be seen in Table 7.

### 5.6. Comparison of Creep and Shrinkage with Prediction Models

B4 model and B4-TW model were used to predict the creep and shrinkage of the concrete. The B4-TW model extends and refines the B4 model so that the parameter values of the two models are almost the same and are given in [28]. However, the B4 model used a ratio of water to cement to calculate the parameters and is adjusted to improve the prediction for concrete using mineral admixtures, whereas the B4-TW model used a water to cementitious materials ratio to directly determine the values of the parameters. Additionally, the B4-TW model extends new parameters in the formulae of creep and shrinkage.

Z. P. Bažant [38] indicated that the values of creep and shrinkage are influenced by many factors which may be divided into intrinsic and extrinsic. In this study, all the intrinsic factors were fixed, including the concrete mix parameters, such as the elastic modulus of aggregate, the type of cement and admixtures, the raw materials content, and the maximum aggregate size, as well as the compressive strength. Hence, the comparison between the B4 model and the B4-TW model is only based on the extrinsic factors, which can be altered after casting, including the loading age, curing time, and relative humidity. Table 8 presents the major creep parameters of two the models and the main different shrinkage parameters of the B4 model and the B4-TW model are listed in Table 9.

#### 5.6.1. Comparison with Prediction Models for Creep

**Figure 13:**
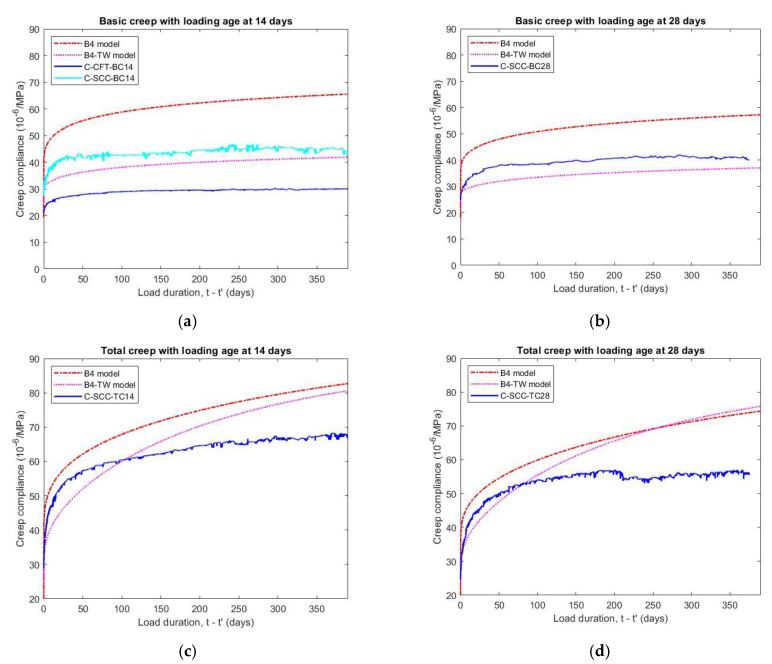
Comparison of experimental creep compliance and prediction models B4 and B4-TW: (**a**) basic creep loaded at 14 days, (**b**) basic creep loaded at 28 days, (**c**) total creep loaded at 14 days, (**d**) total creep loaded at 28 days.

As can be seen in Table 8, the value of compliance parameters *q*_2_, *q*_3_ and *q*_4_, which are factors that affect the basic creep value, predicted by the empirical formula of the B4 model was higher than the value calculated based on the B4-TW model. Therefore, the B4 model gave much higher—from 54 to 57%—long-time values of basic creep compliance than those of the B4-TW model at 375 days after different loading ages, as illustrated in Figure 13a,b. When the specimens were loaded at 14 days and 28 days, the basic creep value of the B4-TW model was also consistent with the experiment data in comparison with the B4 model and the error attained by the B4-TW model was only −7% and −8% for basic creep specimens, respectively.

However, the B4-TW model gave a higher contribution of drying creep than the B4 model because the parameter *q*_5_—that controls the magnitude of the drying creep compliance—calculated by B4-TW model, is 1.84 times higher than for the B4 model. Furthermore, the instantaneous strain *q*_1_, which is closely related to the elastic modulus value, estimated by the B4-TW model, is also larger than that estimated by the B4 model (give in Table 8). These lead to similar predictions for the total creep compliance by both the B4 model and the B4-TW model, as illustrated in Figure 13c,d, although the basic creep compliance of the two models are different. It also can be observed that the prediction values for the models were only consistent with the test data of short-term total creep under 100 days but higher than those of the long-term total creep with various loading ages. The total creep compliance at loading age of 14 and 28 days obtained by B4-TW model is higher than the experimental results by 19% and 34%, while these values of B4 model are 22% and 32%, respectively.

The comparisons between the measured creep compliance of concrete and the prediction values of models B4, B4-TW at 375 days after applied stress are listed in Table 10.

#### 5.6.2. Comparison with Prediction Models for Shrinkage

**Figure 14:**
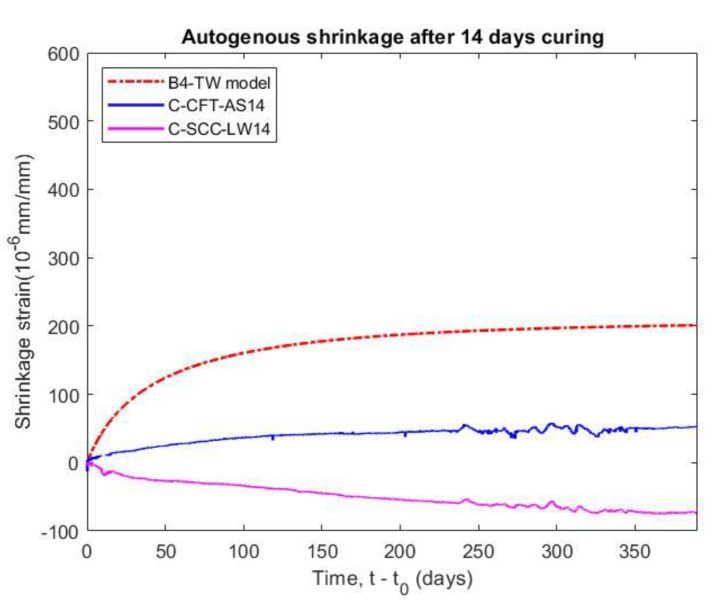
Comparison of experimental results and prediction model B4-TW for autogenous shrinkage measured at 14 days.

The autogenous shrinkage results obtained by the B4-TW model were much higher than the test data for both the confined concrete (C-CFT-AS14 specimen) and the plain concrete curing in moist conditions (C-SCC-LW14 specimen), as observed in Figure 14. Whereas the B4 model does not predict the autogenous shrinkage of concrete with Type II Portland cement (slow hardening) because the final autogenous shrinkage halftime *ε_au__∞_* of B4 model is equal to zero, as given in Table 9.

**Figure 15:**
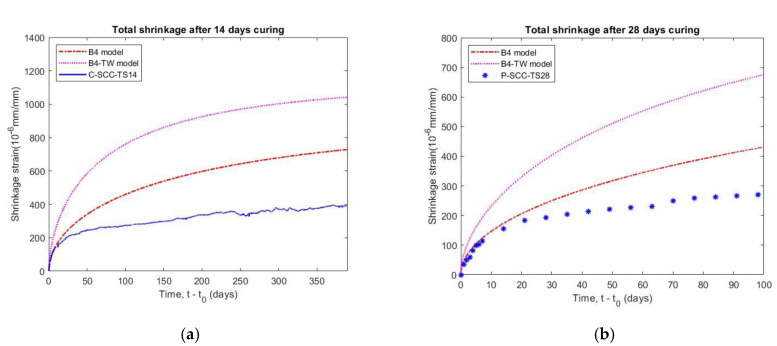
Comparison of measured total shrinkage of SCC with prediction models B4, B4-TW: (**a**) cylindrical specimen cured for 14 days, (**b**) prism specimen cured for 28 days.

The total shrinkage of specimens with a curing age of 14 days (C-SCC-TS14) and 28 days (P-SCC-TS28) are compared to the values of the B4 and B4-TW prediction models, as shown in Figure 15. Both prediction values are overestimated and it implies that the B4 and B4-TW models may not applicable to SCC with expansive additives. The main reason is that the empirical formula of the B4 model does not consider SCC using the slow hardening cement type in autogenous shrinkage. Additionally, the theoretical final shrinkage for drying at zero ambient humidity *ε_sh__∞_* for the B4 model is also less than that of the B4-TW model, as shown in Table 9.

In the study, the SCC was used to fill the steel tube but the shrinkage of the SCC is higher than the plain concrete due to its higher amount of paste and lower water-to-binder ratio. The addition of expansive additive to the SCC in the CFT is common to prevent the gap caused by significant autogenous shrinkage. However, the experiment shrinkage also showed large divergences in comparison with the predicted values of the models, as presented in Table 11. The results indicate that the B4 model and the B4-TW model are inconsistent with the experimental shrinkage of the SCC, especially when using an expansive additive. Therefore, it is necessary to develop an appropriate prediction model to be applied for SCC.

## 6. Conclusions

This paper tests the creep and shrinkage behavior of CFT and concrete without steel tube specimens. The SCC used an expansive admixture to fill a steel tube of 108 mm in diameter, 3 mm in thickness, and 300 mm in length. The time-dependent deformation was performed under different curing conditions over a long time period of up to 400 days. Based on the experimental results, the main conclusions of the study can be drawn as follows:

(1) The advantage of the interaction between the steel tube and the concrete core significantly reduced the time-dependent deformation of CFT. The steel tube has a role as a restraint on the concrete, creating an enclosed environment that prevents the concrete spalling and the relaxing of the impacted stress. Therefore, the creep compliance and shrinkage strain of the CFT specimens was33% and 62%lower than that of the concrete without the steel tube, respectively.
(2) The creep strain in the moisture condition was lower than that in the drying condition. Additionally, the various loading ages that affected the total creep were higher than basic creep because the basic creep is not influenced by the level of humidity, the total creep has a rapid drying process at an earlier age. However, the different curing ages negligibly affect the total shrinkage and length change of the specimens.
(3) The vertical strain of the CFT components had a similar magnitude which was only 1.1% different compared to the vertical strains of the steel tube and the concrete core so they can be considered as fully composite members.
(4) The vertical stress of the steel increased by 32.7% but the vertical stress of the concrete decreased by 15.8% at 375 days. This stress transfer magnitude cannot be neglected and should be considered in CFT design.
(5) The B4-TW model is more consistent than the B4 model in predicting the basic creep of the concrete. The prediction values of the two models were close to the test data of the short-term total creep under about 100 days but higher than those of the long-term total creep. The B4 model does not predict the autogenous shrinkage of concrete with slow hardening cement. Additionally, both the B4 model and the B4-TW model are not suitable to estimate the shrinkage of SCC using expansive admixture.

## Figures and Tables

**Table 1:** Mix proportion of self-compacting concrete (SCC).

Mix Proportion (kg/m^3^)								w/cm	s/a
Cement	GGBS	SF	Sand	CA	Water	SP	EA		
384	256	50	730	789	165	14.3	20	0.24	0.48

**Table 2:** Specimens design for creep and shrinkage tests.

Test Type	Shape	Specimens	Test Age (days)	Dimensions (mm)	Curing Condition	Quantity
Basic creep	Cylinder	C-CFT-BC14	14	108 × 300 × 3	Steel tube	3
Basic creep	Cylinder	C-SCC-BC14	14	Φ100 ×300	Moist	3
Basic creep	Cylinder	C-SCC-BC28	28	Φ100 × 300	Moist	3
Total creep	Cylinder	C-SCC-TC14	14	Φ100 × 300	Drying	3
Total creep	Cylinder	C-SCC-TC28	28	Φ100 × 300	Drying	3
Autogenous shrinkage	Cylinder	C-CFT-AS14	0	108 × 300 × 3	Steel tube	3
Total shrinkage	Cylinder	C-SCC-TS14	0	Φ100 × 300	Drying	3
Total shrinkage	Prism	P-SCC-TS28	28	100 × 100 × 285	Drying	3
Length change	Cylinder	C-SCC-LW14	0	Φ100 × 300	Moist	3
Length change	Prism	P-SCC-LW28	28	100 × 100 × 285	Moist	3

**Table 3:** Compressive strength and elastic modulus of SCC at various days.

Time (days)	7	14	28	56	91
Strength (MPa)	70	76	86	90	92
Elastic modulus (GPa)	29.8	30.7	32.8	32.5	32.8

**Table 4:** Conversion of designated stress ratio to real stress ratio by consideration of VWSG in the specimen.

Specimens	Designated Stress Ratio, *n_c_*	Axial Load *N_cft,c_*(*N_c_*), kN	Elastic Modulus *E_mts_*(*t*′), MPa	Factor *n*(*t*′)	Area *A_e,c_*(*t*′), mm^2^	Stress *σ_c_*(*t*′), MPa	Real Stress Ratio *n_c_*(*t*′)
C-CFT-BC14	0.3	219	44,510	4.4	9132	24.0	0.32
C-SCC-BC14 C-SCC-TC14		180	30,680	6.4	9386	19.2	0.25
C-SCC-BC28 C-SCC-TC28		202	32,770	6.0	9258	21.8	0.25

**Table 5:** Basic and total creep results of specimens.

Specimens	Applied Stress, *σ_c_* (MPa)	Elastic Strain, *ε_e_* (10^−6^)	Creep Values at 375 Days after Loading		
			Creep Strain, *ε_cc_* (10^−6^)	Creep Compliance, *ε_sp_* = (*ε_e_* + *ε_cc_*)/*σ_c_* (10^−6^/MPa)	Creep Coefficient *φ* = *ε_cc_*/*ε_e_*
C-CFT-BC14	24.0	502	218	30.0	0.434
C-SCC-BC14	19.2	540	323	45.0	0.599
C-SCC-TC14	19.2	556	734	67.2	1.321
C-SCC-BC28	21.8	541	331	40.0	0.611
C-SCC-TC28	21.8	549	674	56.1	1.228

**Table 6:** Time-dependent deformation of specimens.

Curing Time, t_0_ (days)	Measured Time, t-t_0_ (days)	Shrinkage Strain		Creep Strain				Total Strain *ε* (10^−6^)
		Unloaded Specimens	Measured Shrinkage *ε_cs_* (10^−6^) at t	Loaded Specimens	Elastic Strain *ε_e_* (10^−6^)	Measured Creep *ε_cc_* (10^−6^)	Real Creep *ε_cr_* (10^−6^)	
			(1)		(2)	(3)	(4) = (3) − (1)	(5) = (1) + (2) + (4)
14	375	C-CFT-AS14	52	C-CFT-BC14	502	218	166	720
14	375	C-SCC-LW14	−73	C-SCC-BC14	540	323	396	863
14	375	C-SCC-TS14	391	C-SCC-TC14	556	734	343	1290
28	98	C-SCC-LW28	43	C-SCC-BC28	541	297	254	838
28	98	C-SCC-TS28	270	C-SCC-TC28	549	625	355	1174

**Table 7:** Vertical strains, stresses and stress transfer of CFT member.

t-t’, Days	Steel Tube			Concrete Core		
	Strain, μ*ε*	Stress, MPa	Stress Transfer, %	Strain, μ*ε*	Stress, MPa	Stress Transfer, %
0	536	107	0.0	502	24.0	0.0
21	630	126	+17.8	642	21.9	−8.8
375	712	142	+32.7	720	20.2	−15.8

**Table 8:** Creep parameters of prediction models B4, B4-TW.

Model	*E_cm28_* (MPa)	The Compliance Parameters (10^−6^/MPa)				
		*q*_1_	*q*_2_	*q*_3_	*q*_4_	*q*_5_
B4	43,900	18.2	65.6	13.3	3.4	194
B4-TW	35,530	22.5	27.1	2.7	2.7	357

**Table 9:** Shrinkage parameters of prediction models B4, B4-TW.

Model	Autogenous Shrinkage		Drying Shrinkage			
	*τ_au_* (days)	*ε_au__∞_* (10^−6^)	*τ*_0_ (days)	*ε_0_* (10^−6^)	*τ_sh_*	*ε_sh__∞_* (10^−6^)
B4	1.45	0	0.067	651	374	1044
B4-TW	-	462	0.031	618	213	1056

**Table 10:** Comparison of measured creep compliance and predicted results at 375 days after loading.

Specimens	Loading Age, t′ (days)	Creep Compliance (10^−6^/MPa)		
		Measured Value, *ε_sp_*	B4 Model	B4-TW Model
C-CFT-BC14	14	30.0	65.5 (+118%)	41.7 (+39%)
C-SCC-BC14	14	45.0	65.5 (+46%)	41.7 (−07%)
C-SCC-TC14	14	67.2	82.0 (+22%)	80.0 (+19%)
C-SCC-BC28	28	40.0	57.1 (+43%)	36.9 (−08%)
C-SCC-TC28	28	56.1	74.0 (+32%)	75.0 (+34%)

**Table 11:** Comparison of measured shrinkage and predicted results.

Specimens	Curing Time, t_0_ (days)	Measured Time, t-t_0_ (days)	Shrinkage Strain (10^−6^ mm/mm)		
			Measured Value, *ε_cs_*	B4 Model	B4-TW Model
C-CFT-AS14	14	375	52	0 (−100%)	202 (+288%)
C-SCC-LW14	14	375	−73	0 (+100%)	202 (+377%)
C-SCC-TS14	14	375	391	723 (+85%)	1040 (+166%)
P-SCC-TS28	28	98	270	427 (+58%)	669 (+148%)

## Data Availability

Data sharing not applicable.
